# Accelerated mutator phenotype in a clinical *Aspergillus fumigatus* isolate contributes to adaptive evolution

**DOI:** 10.1080/22221751.2026.2627078

**Published:** 2026-03-16

**Authors:** Yinggai Song, Margriet W. J. Hokken, Jan Zoll, Hanka Venselaar, Paul E. Verweij, Willem J. G. Melchers, Johanna Rhodes

**Affiliations:** a Department of Dermatology and Venerology, Peking University First Hospital, Beijing, People’s Republic of China; b Department of Medical Microbiology, Radboud Institute for Molecular Life Sciences, Radboud University Medical Center, Nijmegen, the Netherlands; c Radboudumc-CWZ Center of Expertise for Mycology, Nijmegen, the Netherlands; d Research Center for Medical Mycology, Peking University, Beijing, People’s Republic of China; e Department of Medical BioSciences, Radboudumc, Nijmegen, the Netherlands

**Keywords:** *Aspergillus fumigatus*, mutator phenotype, Mre11, antifungal resistance, azole therapy

## Abstract

The opportunistic pathogen *Aspergillus fumigatus* represents a major threat to immunocompromised individuals and is increasingly resistant to antifungal therapies. Resistance selection primarily takes place through environmental selection to azole fungicides, but in-host resistance may develop in patients with chronic aspergillosis receiving azole therapy. In this study, we examine clinical *A. fumigatus* isolates that exhibit irregular growth and accumulated mutations rapidly during antifungal treatment. Whole-genome sequencing of serial isolates revealed an accelerated mutation rate as the likely driver of the observed phenotype. The mutation frequency of this isolate was approximately 15-times higher than other *A. fumigatus* strains. We identified non-synonymous single nucleotide polymorphisms (SNPs) as potential loci involved in the increased mutation rate. Using CRISPR/Cas9 gene editing and comprehensive genomic analysis, we show that a mutation in *mre11*, a gene critical for genomic stability during DNA replication, is responsible for this elevated mutation rate. Mutations within *mre11* result in a 27% reduction in radial growth, highlighting the fitness cost associated with the higher mutation rate. All *mre11*-mutant isolates in this study belong to clade B, a lineage that rarely carries environmental azole-resistance mutations, potentially supporting in-host adaptation. The Phe332Leu allele was observed both in clinical and environmental isolates, suggesting that the mutator phenotype may represent a general adaptive strategy, allowing *A. fumigatus* to persist under prolonged azole pressure. We hypothesize that this heightened mutation background could facilitate the rapid spread of antifungal resistance alleles within *A. fumigatus* populations.

## Introduction

The human fungal pathogen *Aspergillus fumigatus* poses a significant public health threat on a global scale and has emerged as the predominant etiological agent of *Aspergillus*-related diseases worldwide [1,2]. *A. fumigatus* is capable of causing a wide range of diseases in at-risk individuals, and in excess of 2 million people develop invasive aspergillosis (IA) annually in the context of chronic obstructive pulmonary disease, intensive care, lung cancer, or hematological malignancy, with a crude annual mortality of 85.2% [3]. Prior to the introduction of the azole class, resistance to clinical antifungals in *A. fumigatus* was rarely reported; however, rates of resistance to the mold-active azoles are reported as high as 10% [4].

Triazole resistance in clinical *A. fumigatus* isolates is primarily associated with environmental fungicide exposure [5]; indeed, the first isolates with the TR_34_/L98H polymorphism conferring triazole resistance were observed in environmental and clinical isolates, supporting the theory of environmental selection of resistance [6,7]. Spores of azole-resistant *A. fumigatus* strains are easily dispersed from environmental niches through air, travelling long distances before being inhaled by at-risk patients and causing invasive lung infection [8].

Population genomic analyses of *A. fumigatus* have shown pronounced genetic clustering into two highly supported populations, A and B, with the majority of environmentally associated azole-resistance polymorphisms clustering in isolates within population A [5], and azole-susceptible isolates mostly identified in population B. In addition to signature *Cyp51A*-mediated mutations associated with environmental resistance selection (e.g. Tandem Repeat (TR) combined with additional mutations, TR_34_/L98H and TR_46_/Y121F/T289A), isolates within population A were found to have an increased propensity to develop resistance to antifungal compounds because of mutations in the DNA mismatch repair system (MMR) through elevated mutation rates [9].

Elevated mutation rates have been implicated in driving rapid evolution and adaptation in human fungal pathogens, such as *Candida* and *Aspergillus* species [9–12]. creates“mutator” fungi, these elevated mutation rates can confer evolutionary advantages but are often associated with fitness costs when deleterious mutations accumulate. In *A. fumigatus*, the pervasive use of agricultural azoles has led to the selection of mutator fungi that harbour the *cyp51A* triazole resistance allelic variant TR_34_/L98H [9,10]. This has resulted in a lineage of *A. fumigatus* that is not only multi-azole resistant but also multi-fungicide resistant, with strong selection pressure influencing adaptation. However, fungi can also utilize this strategy to adapt successfully and rapidly to other environmental challenges, such as temperature and pH changes, to generate phenotypic variability.

To investigate the genetic basis of in-host adaptation and the early steps preceding resistance evolution, we focused on a series of isogenic, azole-susceptible isolates obtained from a X-linked chronic granulomatous disease (CGD) patient undergoing prolonged azole therapy [13]. Analysis of these strains displayed a varying morphology and sectors within the mycelium. Here, we identify a novel variant in *mre11*, encoding a core component of the MRX-mediated double-strand break repair pathway in *A. fumigatus*, and characterize the corresponding elevated mutation rates.

## Methods

### Culture and morphology of clinical isolates

Three azole-susceptible *A. fumigatus* clinical isolates (V130-14, V147-03, V155-40) were cultured from a patient with CGD who was receiving long-term azole prophylaxis between Nov 25, 2011, and Oct 23, 2013 at Radboud University Medical Centre, Nijmegen, the Netherlands (Table 1), they represent the azole-susceptible baseline population prior to the subsequent emergence of resistant isolates in the same patient [14]. Isolates were cultured on Sabouraud Dextrose Agar (SDA) (Oxoid™) (1% peptone, 4% glucose, 1.5% agar, pH 5.6) or in a tissue culture flask with a semi-permeable lid (Nunc™ EasYflask, ThermoScientific™, Massachusetts, USA) with Aspergillus Minimal Medium (AMM) 1.5% agar containing per liter: (10 g glucose, 5.95 g NaNO_3_, 0.522 g KCl, 1.5 g KH_2_PO_4_, 50 mg MgSO_4_·7H_2_O, 1 mL trace elements). Trace elements contained (per 200 mL): 10 g EDTA, 4.4 g ZnSO_4_·7H_2_O, 1.01 g MnCl_2_·4H_2_O, 0.315 g CuSO_4_·5H_2_O, 0.22 g (NH_4_)6Mo7O_2_·4H_2_O, 1.0 g Fe (II)SO_4_·7H_2_O, and 2.2 g H_3_BO_3_. All compounds were produced by Merck (Darmstadt, Germany). Isolates were stored in 20% glycerol at −80°C, and subcultured on SDA at 37 °C for 4–5 days. Conidia were harvested with a wet cotton swab and resuspended in Milli-Q containing 0.1% Tween 20. Approximately 2.6 × 10^5^ CFU/ mL (Colony Forming Unit) of conidia were used to inoculate each fresh tissue culture flask. All growth and morphological assays on solid media were performed with three independent biological replicates.

**Table 1.** Three azole susceptible clinical isolates were used for serial passaging on AMM for 10 weeks [14].

Isolate	Date of isolation	ICZ	VCZ	PCZ
V130-14	25/11/2011	1	1	0.25
V147-03	25/03/2013	0.5	1	0.25
V155-40	23/10/2013	1	1	0.25

Note: ICZ = itraconazole; VCZ = voriconazole; PCZ = posaconazole.

To assess the possibility of isolate V130-14 being a mutator [15], approximately 2.6 × 10^5^ conidia per isolate were spotted and grown on solid AMM supplemented with 0.02% Methyl Methane Sulfonate (MMS) (Merck Millipore, USA), and were grown at 37 °C for 4 days. Isolates were grown in triplicate on AMM as a control. Colony width was measured after 4 days and compared to control growth of *A. fumigatus* CEA10.

### Serial passaging of clinical isolates and microsatellite genotyping

We conducted a serial passaging experiment in triplicate with the potential mutator isolate V130-14 and two other isolates from the same patient (V147-03 and V155-40) that were azole susceptible, to investigate if the potential mutator isolate V130-14 would accumulate more mutations over time. After 7 days of growth, conidia from the outer edge of the mycelium were transferred with a wet Q-tip to a new tissue culture flask containing 20 ml AMM 1% agar. After 10 weeks, isolates were harvested, grown for 24 h in an Erlenmeyer flask containing liquid AMM. Genomic DNA was prepared from mycelial bulbs as described previously [16] by using phenol/chloroform/isopropanol. As a control, Short Tandem Repeat (STR) analysis was performed to confirm isogeneity of T = 0 and T = 10 isolates. Multiplex PCR was performed for *A. fumigatus* STR loci; three trinucleotide (STRAf3A-C) and three tetranucleotide loci (STRAf4A-C) were amplified using corresponding primers containing the dye carboxyfluorescein (FAM), hexachlorofluorescein (HEX), or tetrachlorofluorescein (TET) at the 5’ end, respectively. Cleaned PCR products were analyzed together with a GeneScan500 LIZ size standard (Applied Biosystems™, UK) and fluorescence was detected on a Genetic Analyzer (Applied Biosystems™, USA). The number of repeats was determined using the Peak Scanner Software v1.0 (Applied Biosystems™, USA). PCR primers, fluorescent labels and amplification conditions were exactly as previously described; STR profile interpretation accounted for the known instability of certain markers [17,18].

### Whole genome sequencing of clinical isolates

Genomic DNA was extracted from serial passaging of the three clinical isolates from the same patient, V130-14, V147-03 and V155-40 [16]. For the first analysis of putative mutator isolate V130-14, a DNA library was prepared using the Nextera XT® DNA sample preparation kit (Illumina, USA). Subsequent sequencing was conducted in a paired end 2 × 150 bp mode using an Illumina NextSeq 500® machine (Illumina, USA) at the Department of Genetics in Radboudumc (Nijmegen, Netherlands). For the WGS analysis of these three azole susceptible isolates the serial passaging experiments were resequenced at BGI genomics (Hong Kong) and sequenced on the BGIseq-500® sequencing platform in a 2 × 100 bp paired-end mode.

Raw sequencing reads were subjected to quality control using FastQC (v0.11.9) and adapter trimming/quality filtering was performed using Trimmomatic (v0.39) with parameters: ILLUMINACLIP:2:30:10, LEADING:3, TRAILING:3, SLIDINGWINDOW:4:15, MINLEN:36.

Mapping of all quality-filtered reads and SNP calling were performed using CLC genomics workbench® V19-22 (Qiagen). Mapping was performed using the “Map Reads to Reference” tool, and reads were mapped against the Af293 ENSEMBL CADRE 30 reference genomes. SNPs were identified using the “Low Frequency Variant Detection tool with the following parameters: Required significance (%) = 1.0, Ignore positions with coverage above = 100.000, Restrict calling to target regions = Not set, Ignore broken pairs = Yes, Ignore non-specific matches = Reads, Minimum coverage = 10, Minimum count = 2, Minimum frequency (%) = 80.0 (This threshold was set to detect mutations that had become fixed or reached high frequency in the V130-14 population by passage T = 10, distinguishing them from low-frequency background variants or sequencing errors), Base quality filter = No, Read direction filter = No, Relative read direction filter = Yes, Significance (%) = 1.0, Read position filter = No, Remove pyro-error variants = No. To identify mutations that arose specifically during the serial passaging, SNPs were called by comparing the T = 10 sample against the baseline T = 0 sample of the same isolate V130-14.

Given the observed MMS sensitivity of V130-14 (Figure 2), indicative of a DNA repair defect, we focused our subsequent analysis on Gene Ontology “Biological Process’, “cellular component” and “molecular function” with the words “repair”, “polymerase”, “nuclease” and “helicase”. The total list of 292 unique amino acid changes that were unique to the putative mutator V130-14 was filtered. Normalized coverage was calculated for each of the three isolates.

### Calculations determining the mutational rate

To correct for growth speed, which differed between isolates, we made an estimation of the total number of cells produced by measuring colony growth every 7 days and by measuring the approximate length of a hyphal cell. Hyphal cells were stained with BlankoPhor and observed through fluorescence microscopy. Mutational rate was determined by the number of mutations per generation per base pair and calculated by the amount of SNPs/number of cells/genome size (29.4 MB; Supplementary Table 1).

### Creation of a protein 3D model

Modelling and calculation of a 3D protein model of the wild-type and mutated protein of AFUA_6G11410 (*mre11*) were completed in CLC genomics workbench**®**, by creating an alignment to the template structure of the catalytic domain of MRE11 (PDB ID: 4YKE) from *Chaetomium thermophilum*, the only available crystal structure of this protein from a filamentous fungus, as the crystal structure of AfMRE11 has not been elucidated. In addition, Mre11 structures were constructed using AlphaFold for *A. fumigatus* Mre11 wild-type and Mre11 F332L [19,20]. Yasara (version 25.1.13) was also used to construct alignments for *A. fumigatus* Mre11 structures and X-ray Mre11 structure from other related fungal species *S. pombe, S. cerevisiae, and T thermophila* [21].

### CRISPR-Cas9 gene modification

A Phe332Leu (F332L) mutation in *mre11*, a highly conserved amino acid residue, was introduced into the *A. fumigatus* wild-type strain A1160^+^, a CEA10-derived laboratory strain deficient in *ku80*. The hygromycin resistance cassette, amplified from plasmid pAN7.1, was used as a selectable marker. crRNAs and repair template oligonucleotides were designed using the web-based guide RNA design tool EuPaGDT [22] under default settings to target the *mre11* locus. crRNAs with the highest QC scores and closest proximity to the intended integration site were manually selected for transformation experiments (Supplementary Table 2). Homology-directed repair (HDR) templates flanked by microhomology arms were amplified by PCR from the mutator strain V130-14 using Phusion Flash Master Mix (Thermo Fisher Scientific) and corresponding primers (Supplementary Table 2). The resulting amplicons were gel-purified using the Qiagen PCR purification kit and used directly for transformation. All gRNAs and primers employed in the CRISPR-Cas9 transformation are listed in Supplementary Table 2. Ribonucleoprotein (RNP) complexes were assembled with Alt-R S.p. Cas9 Nuclease V3, Alt-R® CRISPR-Cas9 tracrRNA, and locus-specific Alt-R® CRISPR crRNA (Integrated DNA Technologies) by heating at 95 °C for 5 min, followed by gradual cooling to room temperature over 10 min in a thermal cycler [23].

The transformation protocol was adapted from van Rhijn et al. and Zhao et al. [23,24]. The transformation mixture was plated onto Sorbitol Minimal Medium (AMM supplemented with 1 M sorbitol and 1.5% [w/v] agar) containing hygromycin B (150 µg/ml). Plates were initially incubated at room temperature for 24 h, then transferred to 37 °C for 48–72 h. Spores from purified colonies were harvested, and genomic DNA was extracted for PCR validation of cassette integration using Phusion Flash Master Mix and relevant primers (Supplementary Table 2), as described previously [16]. PCR products were analyzed by gel electrophoresis, and three independent isolates were subjected to Sanger sequencing. The sequenced genomic regions and the primers used are listed in Supplementary Table 2. Transformation efficiency was calculated as the percentage of PCR-positive strains among all screened transformants. Reversion of the F332L mutation in the putative mutator strain V130-14 was performed using a wild-type repair template following the same procedure.

### Growth testing assays and serial passaging of recombinant genome analysis

Radial growth rates of *mre11*-F332L transformants were conducted by spotting 10^3^ spores per strain on AMM agar plates. Plates were incubated for 6 days, and the radius of the colonies was measured every 24 hours. Three biological replicates were conducted for each strain. Growth measurements of the recombinants were compared to the parental isolate.

*mre11*-F332L transformants and wild-type transformants were cultured on AMM and AMM supplemented with 0.1 µg/ml itraconazole (ICZ) for 10 weeks, and genomic DNA was extracted as previously described [16]. Genome sequencing was performed using high-quality DNA samples (OD 260/280 = 1.8-2.0, > 10 µg) on the Illumina HiSeq platform (Novogene, Beijing, China).

Paired-end reads were aligned using the Burrows–Wheeler Aligner v0.7.17 [25] MEM to the Af293 reference genome GCF_000002655.1 (ASM265v1). Post-processing was conducted using SAMtools v1.16.1 [26] and Picard v2.27.4 [27], and variant calling was performed using GATK HaplotypeCaller v4.2 [28,29], excluding repetitive regions (identified using RepeatMasker v4.0.6) [30], generating GVCFs. Low-confidence variants were labelled as such if they met at least one of the parameters QD < 2.0, FS > 60.0, DP < 5, GQ < 50, MQ < 40, MQRankSum <−12.5, ReadPosRankSum <−8.0, SOR > 4.0. In addition, alternate variants must be present in at least 90% of reads covering that position. Variant annotation and functional effect were completed using snpEff v5.1 [31].

## Results

### Mycelial morphology and growth phenotypes of clinical isolates

**Figure 1.**
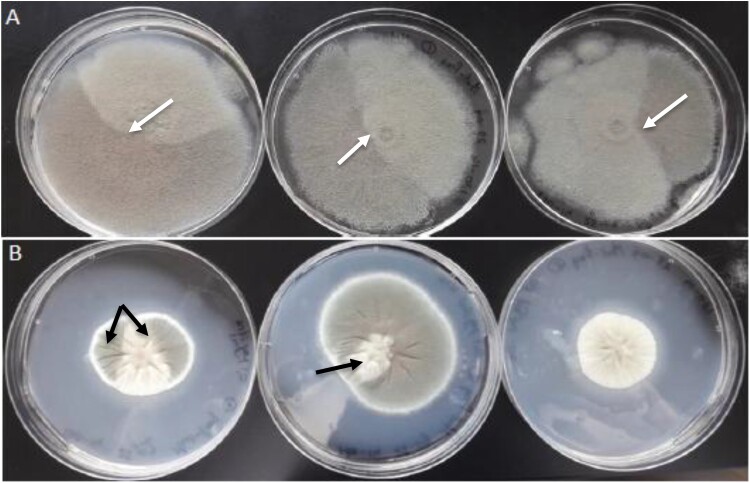
Morphology of clinical isolate V130-14 in triplo.

**Figure 2.**
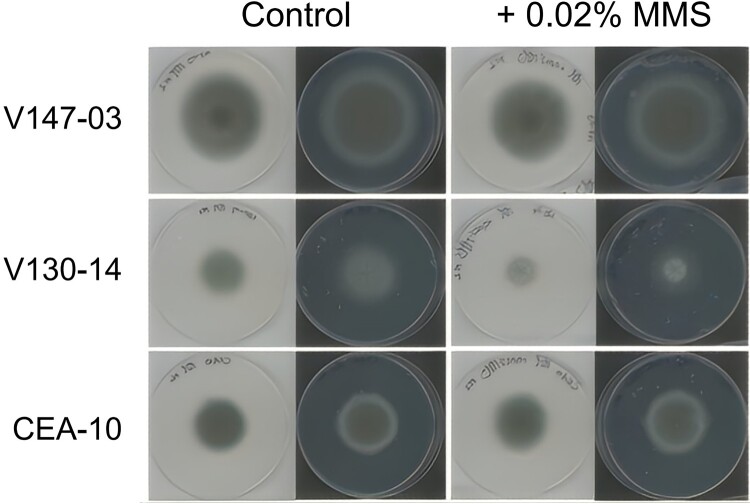
Typical growth of three *A. fumigatus* isolates on AMM and on AMM supplemented with 0.02% MMS. The same plate is shown on a white and on a black background.

This study includes three isolates (V130-14, V147-03 and V155-40) from a single patient, where the morphology of an azole-susceptible isolate (V130-14) on AMM plates revealed phenotypic variability, often characterized by distinct sectors within the mycelium. This was accompanied by discoloration of the conidia and a significant reduction in both radial growth (40% decrease) and conidiation (approximately 60% decrease) compared to the reference strain CEA10 (Figure 1). V130-14 also displayed a growth impairment on MMS, experiencing a 27% reduction in mycelial growth relative to the control (Figure 2). In comparison, V147-03 exhibited a growth ratio of 0.92, and the reference strain CEA10 exhibited a growth ratio of 1.01. While MMS sensitivity is consistent with a DNA repair defect and a potential mutator phenotype, we note that it could also reflect other adaptive processes, such as altered responses to host-derived oxidative stress. We therefore sought to understand the genetic basis of the phenotypic variability displayed by V130-14.

### Calculation of mutation rate and accumulated SNPs over a ten-week period through serial passaging

To approximate the mutation rate in the phenotypically variable isolate V130-14 and two other clinical isolates (V147-03 and V155-40), an estimation was made of the number of generations that passed between timepoints T = 0 weeks and T = 10 weeks. After ten weeks, STR analysis showed the three isolates were still isogenic to the original inoculum (Supplementary Table 3) ruling out contamination during the passaging experiments. We approximated the mutation rate by dividing the total radial growth per ten weeks by the average length of a hyphal cell. We found that the average length of a hyphal cell was 44 µm, which corresponds to 23 cells per mm (Supplementary Figure 1). We found an average mutation rate of 5.49^−11^ for V130-14, and 3.73^−12^ for wild-type isolate V147-03. Due to the lack of mutations found in isolate V155-40, it was not possible to calculate a mutation rate for this isolate (Supplementary Table 1). This data suggests that the mutation rate of isolate V130-14 is approximately 15 times higher than in wild-type isolate V147-03. Therefore, we proposed that V130-14might possess an elevated mutation rate and a mutator phenotype.

After comparing all isolates of T = 10 to their T = 0 original inoculum, a total of 102,570,244 sequence reads were obtained, with an average unique mapping percentage of 98.1% to the reference genome Af293, and an average coverage of 59.11x (Table 2). We found on average 12 SNPs for the mutator isolate V130-14, 0.33 SNPs for isolate V147-03 and 0 SNPs for isolate V155-40 after ten weeks of serial passaging (Table 3). Whereas only 1 SNP was found in the progeny of V147-03, the other 36 SNPs were found in the progeny of putative mutator isolate V130-14, confirming that this isolate is indeed a mutator isolate.

**Table 2.** Mapping results and coverage calculations.

Isolate	Timepoint	# of reads	# of uniquely mapped reads	% of mapped reads	Coverage
V130-14	T = 0	8.144.108	7.877.323	96.72	54.70
V130-14-R1	T = 10	9.538.322	9.421.213	98.77	65.43
V130-14-R2	T = 10	9.416.724	9.301.258	98.77	64.59
V130-14-R3	T = 10	8.029.006	7.928.114	98.74	55.06
V147-03	T = 0	8.161.422	8.007.741	98.11	55.61
V147-03-R1	T = 10	9.562.998	9.399.991	98.29	65.28
V147-03-R2	T = 10	9.583.926	9.405.596	98.13	65.32
V147-03-R3	T = 10	8.022.818	7.883.839	98.26	54.75
V155-40	T = 0	9.585.250	9.391.427	97.97	65.22
V155-40-R1	T = 10	8.129.532	7.965.578	97.98	55.32
V155-40-R2	T = 10	8.144.218	7.975.049	97.92	55.38
V155-40-R3	T = 10	8.172.148	8.013.115	98.05	55.65
CEA10	-	8.151.018	7.916.266	97.12	59.02
V34-78	-	7.986.954	7.857.552	98.38	54.57
V48-46	-	9.542.894	8.751.341	91.71	60.77

**Table 3.** SNPs and corresponding amino acid changes counted after 10 weeks of serial passaging.

Isolate	# SNPs	# AA changes
V130-14-R1	14	7
V130-14-R2	15	4
V130-14-R3	7	3
V147-03-R1	1	0
V147-03-R2	0	0
V147-03-R3	0	0
V155-40-R1	0	0
V155-40-R2	0	0
V155-40-R3	0	0

### Genome analysis of mutator isolate v130-14

In total, we found 56,472 mutations in the genome compared to reference Af293. We filtered mutations that were also present in other isolates, which therefore cannot be causal mutations for the potential mutator phenotype seen in this isolate, to identify unique SNPs. SNPs and indels were filtered against five other genomes that were sequenced in this experiment, V147-03, V155-40, CEA10, and two additional clinical isolates V34-78 and V46-48 (Table 2).

In total, we found 1837 unique mutations, consisting of 70 deletions, 45 insertions, 101 multi nucleotide variances, 8 replacements and 1613 SNPs. Of these mutations, 292 were found to be missense mutations conferring amino acid changes. We observed 15 missense SNPs and indels in 14 genes proven or predicted to be involved in DNA damage repair (Table 4). One of these SNPs, a T994C mutation in *mre11*, resulted in a Phe332Leu substitution; *mre11* is a protein that is part of the highly conserved Mre11-Rad50-Xrs2 (MRX) complex, which plays an important role in double-strand break repair and telomere stability [32].

**Table 4.** List of 14 candidate genes with unique amino acid changes in genes that are predicted or proven to be involved in DNA mismatch repair.

Gene	Name	SNP	AA change	Product description
Afu3g13260	-	1967C > T	Ser656Leu	Has domain(s) with predicted nuclease activity and a role in DNA repair
Afu4g03020	-	421G > T	Val141Leu	Ortholog(s) have a role in chromatin remodeling and the Ino80 complex, cytosol localization
Afu4g06490	*mih3*	490C > T	Pro164Ser	Ortholog(s) have a role in meiotic mismatch repair, reciprocal meiotic recombination and the MutLgamma complex, nucleus localization
Afu5g02570	-	9798G > T	Gln3266His	Ortholog(s) have histone acetyltransferase activity
Afu5g06260	*ino80*	3012G > T	Met1004Ile	Chromatin-remodeling ATPase INO80, putative
Afu6g11410	*mre11*	994T > C	Phe332Leu	Ortholog(s) have manganese ion binding, nuclease activity
Afu6g13290	-	917G > A	Gly306Asp	SNF2 family helicase/ATPase, putative
Afu5g01600	-	1268C > G	Thr423Ser	Has domain(s) with predicted DNA binding, DNA-directed DNA polymerase activity, catalytic activity and a role in DNA replication
Afu5g06590	-	2831C > T	Thr944Met	The domain(s) have predicted ATP binding, DNA binding, helicase activity, hydrolase activity, acting on acid anhydrides, in phosphorus-containing anhydrides and nucleic acid binding, more
Afu5g06600	-	2336G > A, 298G > A	Arg779His, Val100Met	Has domain(s) with predicted ATP binding, DNA binding, helicase activity, nucleic acid binding activity
Afu8g04740	-	5795G > A	Arg1932His	Ortholog(s) have a role in spliceosomal conformational changes to generate catalytic conformation and U5 snRNP, cytosol, spliceosomal complex localization
Afu4g07290	-	442A > G	Arg148Gly	Ortholog(s) have Y-form DNA binding, crossed form four-way junction DNA binding, crossover junction endodeoxyribonuclease activity, flap-structured DNA binding activity and a role in the mitochondrial DNA metabolic process
Afu5g04010	*sen2*	772G > A	Glu258Lys	Putative tRNA-splicing endonuclease subunit
Afu5g04070	*spo11*	1075A > C	Met359Leu	Ortholog(s) have a role in meiotic DNA double-strand break formation, reciprocal meiotic recombination, synapsis and cytosol, nucleus localization

No SNPs causing known mutations that confer resistance to azole antifungal drugs were present in *Cyp51A*. Two missense SNPs were present in only isolates V130-14 and Fa1_1 (recombinant with mre11-F332L); one missense SNP caused Phe35Leu (F35L) in AFUA_1G00950, encoding a protein of unknown function, and the other missense SNP caused Phe332Leu (F332L) in AFUA_6G11410 (*mre11*).

Analysis of publicly available *A. fumigatus* genomes determined that 22 clade B isolates were found to contain this F332L-*mre11* mutation, with seven of these isolates also being from environmental sources (Table 5). The clinical isolate in this study harbouring F332L-*mre11* belongs to clade B, which rarely contains isolates with signature environmental resistance mutations commonly associated with the clade A population. All three susceptible isolates in this study were also *Cyp51A* wildtype (and present on the Clade B background), with ICZ susceptible MIC (Minimum Inhibitory Concentration) data, whereas later isolates from the same patient were itraconazole, voriconazole and/or posaconazole resistant, which contained P216L, G54R, G54 V and M220R, and had an MIC of 4 mg/l or >16 mg/l for ICZ [13].

**Table 5.** Metadata associated with Clade B isolates from publicly available *A. fumigatus* genomes also found to contain the *mre11* F332L mutation responsible for an accelerated mutation rate, and mutator isolate identified in this study.

Isolate ID	Country	Year	Source	ICZ MIC (if known)	Accession
C16	Japan	2009	Clinical		PRJDB1541
C18	Japan	2009	Clinical		PRJDB1541
C416	Japan	2009	Clinical	1	PRJDB3064
C417	Japan	2009	Clinical	1	PRJDB3064
C58	Ireland	2010	Clinical	0.12	PRJEB27135
C59	Ireland	2010	Clinical	0.06	PRJEB27135
C418	Japan	2011	Clinical	4*	PRJDB3064
C72	Ireland	2011	Clinical	0.12	PRJEB27135
C73	Ireland	2011	Clinical	0.03	PRJEB27135
C505	USA	2015	Clinical		PRJNA632561
C531	USA	2015	Clinical		PRJNA632561
C532	USA	2015	Clinical		PRJNA632561
E427	USA	2015	Environment		PRJNA632561
E429	USA	2015	Environment		PRJNA632561
C446	USA	2016	Clinical		PRJNA632561
C470	USA	2016	Clinical		PRJNA632561
C481	USA	2016	Clinical		PRJNA632561
E594	USA	2017	Environmental	1	PRJNA742769
E606	USA	2017	Environmental	1	PRJNA742769
E372	UK	2018	Environmental		PRJEB51237
E377	UK	2018	Environmental		PRJEB51237
E547	USA	2018	Environmental	1	PRJNA742769
V130-14	Netherlands	2011	Clinical	1	PRJEB63121

Note: ICZ = itraconazole. *caused by P216L in *Cyp51A*.

A. Growth on AMM for 4d. B. Growth on AMM supplemented with 0.1 µg/ml ICZ for 4d. (Sectors were shown in arrows).

### Protein 3D model

**Figure 3.**
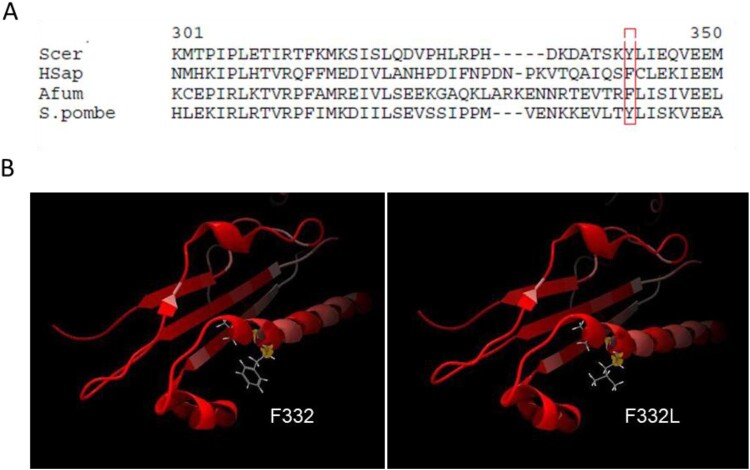
Alignment and predicted protein structure of wildtype and mutated MRE11 in *A. fumigatus*. Position 332 is located on the outside of an alpha helix.

Sequence alignment of this region of the gene for *A. fumigatus*, *S. pombe*, *S. cerevisiae* and *H. sapiens* shows a highly conserved tyrosine or phenylalanine at this location for all species (Figure 3A). We constructed a 3D model of the mutation based on the crystal structure of MRE11 from *S. cerevisiae* for the wildtype and the mutated sequence of MRE11. The F332L mutation results in a change on the surface of the MRE11 interface, as the aromatic phenylalanine is replaced by leucine (Figure 3B). Additionally, alignments for *A. fumigatus* Mre11 wild-type and Mre11 F332L were constructed using the EMBL-EBI MAFFT-tool (https://www.ebi.ac.uk/Tools/msa/mafft/). Both the phenylalanine and leucine fit into the alpha-helical structure, suggesting the mutation does not cause significant structural differences.

**Figure 4.**
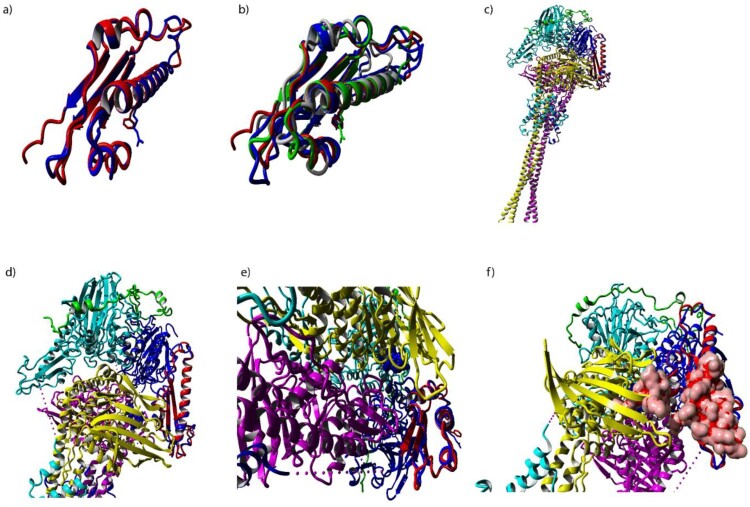
Figures created in Yasara a) Alignment of *A. fumigatus* (red) and *T. thermophila* (blue) MRE11. The animo acid side-chain of residue 332 is shown. b) Alignment of five MRE11 structures c) *C. themophila* Mre11-Rad50-Nbs1 complex aligned with the region of interest (red) d) Alignment of *C. thermophila* region of interest (red) in the Mre11 complex (blue) e) Zoomed in view of alignment of *C. thermophila* region of interest (red) in the Mre11 complex (blue) f) *C. thermophila* Mre11-Rad50-Nbs1 complex with the region of interest.

RMSD values were calculated using Yasara (Figure 4). RMSD values of *A. fumigatus* Mre11 versus Mre11 structures from *T. thermophila*, *S. pombe*, and *S. cerevisiae* were 0.725, 0.938, and 1.011 Å respectively.

### Morphology and growth testing of *mre11*-F332L transformants

The *mre11*-F332L transformants were subjected to detailed morphological and growth assessments. Whole-genome sequencing of the *mre11*-F332L transformants and the original mutator isolate V130-14 (10-day passage experiment, ≥200× coverage) identified 295 unique SNPs genome-wide. Of these, only three were located within protein-coding genes, and only two caused non-synonymous changes. The F332L substitution in Mre11 was the most recurrent and is therefore the candidate driver of the elevated mutation rate.

The growth impact of the *mre11*-F332L mutation was less pronounced than that of the original mutator strain V130-14, and did not reach statistical significance (Supplementary Figure 2). No sectors were observed in the *mre11*- F332L transformants, which is consistent with the original mutator strain, where sector formation was not always present.

## Discussion

We investigated the mutation rate of a putative mutator isolate through WGS and found that it had a 15-fold increase in SNPs that persisted in the population after ∼7000 generations compared to wild-type strains. The average mutational rate found for isolate V130-14 (5.49^−11^) is five times higher than the mutational rate found in an *A. fumigatus* mutator isolate recently studied, which had a mutation rate of approximately 1.1 × 10^−11^ [33]. The radial growth of the putative mutator isolate was diminished by 27% compared to the control strain, whereas wild-type isolate V147-03 only had a reduction of radial growth by 8%. These results suggest a fitness defeat associated with elevated mutation rates and are similar to the growth reduction found in literature, which shows a growth reduction in *A. fumigatus* isolates with diminished genetic stability, as compared to the wild-type [10,34].

We identified a T994C mutation in the *mre11* gene of V130-14, resulting in a Phe332Leu (F332L) substitution. The *mre11* gene encodes a protein that is a component of the highly conserved Mre11-Rad50-Xrs2 (MRX) complex, which plays a crucial role in double-strand break repair and telomere stability [35]. However, we did not find any SNPs in gene *mre11* in any of the other five sequenced isolates when compared to the reference genome of Af293, which was not the case for any other candidate gene. A mutation in *mre11* gene could have consequences on the functionality of this complex: a previous study confirmed that a Phe328Leu mutation, which is a similar amino acid change in a nearby codon, causes a growth defect on medium supplemented with MMS in *S. cerevisiae* [36]. This mutation destabilizes the binding of MRE11p with RAD50p, resulting in a defect in DNA damage repair. The 3D model indicates that this region of the gene contains a highly conserved tyrosine or phenylalanine at this location across all species. The crystal structure of MRE11 from *S. cerevisiae* shows that position 332 is located on the outside of an alpha helix that forms a bonding interface with RAD50. As the aromatic phenylalanine is replaced by leucine, it is possible that this mutation also leads to decreased binding of AfMRE11 to AfRAD50 in *A. fumigatus*, thereby causing a defect in DNA double-strand break repair. Future studies are needed to directly test the mechanistic impact of the F332L mutation on MRX complex stability and nuclease activity in *A. fumigatus*.

In our study, we aimed to validate the observed increase in SNPs by restoring the wildtype MRE11 locus through CRISPR/Cas9 transformation in the same genetic background. The results indicated that the mre11-F332L variant was largely sufficient to explain the elevated mutational rate, confirming its causative role in the mutator phenotype. Additionally, the mutation had a slight but measurable effect on the growth rate, indicating that while the mutator phenotype is primarily characterized by an increased mutational rate, it also has subtle effects on growth dynamics. The absence of sector formation, while informative, is not always a consistent feature in the original strain, suggesting that other genetic or environmental factors may influence this phenotype.

The clinical isolate harbouring the *mre-11* mutation belongs to clade B, a clade that rarely possesses triazole resistance mutations. Recently, elevated mutation rates were reported for (clade A) isolates harbouring TR_34_/L98H, likely exposed to agricultural azole fungicides [9]. Together with our finding of the F332L allele in both clinical and environmental isolates, we hypothesize that the mutator genotype represents a general adaptive strategy in *A. fumigatus* to withstand prolonged azole stress. Although our investigation originated from a detailed analysis of a single patient's series, the identification of the *mre11* F332L allele in a broader set of clinical and environmental Clade B isolates elevates its significance. It suggests this mutator genotype could be a conserved mechanism that enhances the evolutionary potential of *A. fumigatus* populations facing sustained antifungal pressure, both in clinical and environmental settings. This strategy may extend beyond drug resistance to encompass broader host adaptation, as hypermutation could facilitate genetic changes that enhance survival within the dynamic host microenvironment.

The continued selection pressure exerted by the environmental and medical azoles could drive the expansion of these mutator backgrounds in the global *A. fumigatus* populations. This expansion, in turn, leads to opportunities for sexual recombination with isolates containing antifungal resistance alleles, further expanding the genomic variation and complicating the management of drug-resistant disease. Given the high recombination rates inherent in *A. fumigatus*, these resistance polymorphisms are likely to spread rapidly through populations if crossed on to a mutator background [37]. This not only facilitates the acquisition of resistance but also accelerates its dissemination, posing a significant challenge to public health efforts aimed at controlling fungal infections. The mutator background, therefore, represents a critical factor in the evolution of drug resistance in *A. fumigatus*, and understanding its mechanisms and impacts is essential for developing effective strategies to mitigate the spread of resistant strains.

In conclusion, our study highlights the complex interplay between genetic mutations, recombination rates, and environmental factors in the evolution of antifungal drug resistance in *A. fumigatus*. The findings underscore the need for continued research into the genetic and environmental drivers of resistance, as well as the development of novel therapeutic and preventive strategies to address this growing public health concern. Moreover, future investigations should extend beyond single-nucleotide polymorphisms to explore whether mutator phenotypes also affect the variation dynamics of STRs. Elevated STR variation in regulatory or coding regions of genes such as *cyp51A* could constitute an alternative and rapid pathway to antifungal resistance. Concurrently, although the *mre11*-F332L allele elevates the basal mutation rate, its specific contribution to accelerating the acquisition of resistance-conferring mutations in *cyp51A* remains to be empirically established, representing a critical direction for future work.

## Supplementary Material

Supplementary materials 12_12_2025.pdf

MRE11.xlsx

## Data Availability

All raw reads have been submitted to the European Nucleotide Archive (ENA) under accession number PRJEB63121.

## References

[1] Latgé JP, Chamilos G. *Aspergillus fumigatus* and Aspergillosis in 2019. Clin Microbiol Rev. 2019;33:e00140-18. doi:10.1128/CMR.00140-18 31722890 PMC6860006

[2] Verweij PE, Rijnders BJA, Brüggemann RJM, et al. Review of influenza-associated pulmonary aspergillosis in ICU patients and proposal for a case definition: an expert opinion. Intensive Care Med. 2020;46:1524–1535. doi:10.1007/s00134-020-06091-6 32572532 PMC7306567

[3] Denning DW. Global incidence and mortality of severe fungal disease. Lancet Infect Dis. 2024;24:e428–e438. doi:10.1016/S1473-3099(23)00692-8 38224705

[4] Buil JB, Snelders E, Denardi LB, et al. Trends in azole resistance in *Aspergillus fumigatus* , The Netherlands, 1994-2016. Emerg Infect Dis. 2019;25:176–178. doi:10.3201/eid2501.171925 30561296 PMC6302600

[5] Rhodes J, Abdolrasouli A, Dunne K, et al. Population genomics confirms acquisition of drug-resistant *Aspergillus fumigatus* infection by humans from the environment. Nat Microbiol. 2022;7:1944. doi:10.1038/s41564-022-01160-6 36203089 PMC9613473

[6] Verweij PE, Mellado E, Melchers WJ. Multiple-triazole-resistant aspergillosis. N Engl J Med. 2007;356:1481–1483. doi:10.1056/NEJMc061720 17409336

[7] Verweij PE, Snelders E, Kema GH, et al. Azole resistance in *Aspergillus fumigatus*: a side-effect of environmental fungicide use? Lancet Infect Dis. 2009;9:789–795. doi:10.1016/S1473-3099(09)70265-8 19926038

[8] Kanj A, Abdallah N, Soubani AO. The spectrum of pulmonary aspergillosis. Respir Med. 2018;141:121–131. doi:10.1016/j.rmed.2018.06.029 30053957

[9] Bottery MJ, van Rhijn N, Chown H, et al. Elevated mutation rates in multi-azole resistant *Aspergillus fumigatus* drive rapid evolution of antifungal resistance. Nat Commun. 2024;15:10654. doi:10.1038/s41467-024-54568-5 39681549 PMC11649685

[10] Dos Reis TF, Silva LP, de Castro PA, et al. The *Aspergillus fumigatus* mismatch repair MSH2 homolog is important for virulence and azole resistance. mSphere. 2019;4:416–419. doi:10.1128/mSphere.00416-19 PMC6686229 31391280

[11] Healey KR, Zhao Y, Perez WB, et al. Prevalent mutator genotype identified in fungal pathogen *candida glabrata* promotes multi-drug resistance. Nat Commun. 2016;7:1–10. doi:10.1038/ncomms11128 PMC5603725 27020939

[12] Vale-Silva L, Beaudoing E, Tran VDT, et al. Comparative genomics of two sequential candida glabrata clinical isolates. G3 (Bethesda). 2017;7:2413–2426. doi:10.1534/g3.117.042887 28663342 PMC5555451

[13] Ballard E, Melchers WJG, Zoll J, et al. In-host microevolution of *Aspergillus fumigatus*: A phenotypic and genotypic analysis. Fungal Genet Biol. 2018;113:1–13. doi:10.1016/j.fgb.2018.02.003 29477713 PMC5883321

[14] Hokken MWJ, Zoll J, Coolen JPM, et al. Phenotypic plasticity and the evolution of azole resistance in *Aspergillus fumigatus*; an expression profile of clinical isolates upon exposure to itraconazole. BMC Genomics. 2019;20:28. doi:10.1186/s12864-018-5255-z 30626317 PMC6327609

[15] Verweij PE, Zhang J, Debets AJM, et al. In-host adaptation and acquired triazole resistance in *Aspergillus fumigatus*: a dilemma for clinical management. Lancet Infect Dis. 2016;16:e251–e260. doi:10.1016/S1473-3099(16)30138-4 27638360

[16] Umesha S, Manukumar HM, Raghava S. A rapid method for isolation of genomic DNA from food-borne fungal pathogens. 3 Biotech. 2016;6:123. doi:10.1007/s13205-016-0436-4 PMC4909022 28330193

[17] de Valk HA, Meis JF, Curfs IM, et al. Use of a novel panel of nine short tandem repeats for exact and high-resolution fingerprinting of *Aspergillus fumigatus* isolates. J Clin Microbiol. 2005;43:4112–4120. doi:10.1128/JCM.43.8.4112-4120.2005 16081958 PMC1233892

[18] de Groot T, Meis JF. Microsatellite stability in STR analysis *Aspergillus fumigatus* depends on number of repeat units. Front Cell Infect Microbiol. 2019;9:82. doi:10.3389/fcimb.2019.00082 30984630 PMC6449440

[19] Jumper J, Evans R, Pritzel A, et al. Highly accurate protein structure prediction with AlphaFold. Nature. 2021;596:583–589. doi:10.1038/s41586-021-03819-2 34265844 PMC8371605

[20] Varadi M, Bertoni D, Magana P, et al. Alphafold protein structure database in 2024: providing structure coverage for over 214 million protein sequences. NAR. 2024;52:D368–D375. doi:10.1093/nar/gkad1011 37933859 PMC10767828

[21] Krieger E, Vriend G. YASARA view. Bioinformatics. 2014;30:2981–2982. doi:10.1093/bioinformatics/btu426 24996895 PMC4184264

[22] Nicolas AG, Serero A, Legoix-Né P, et al. Mutational landscape of yeast mutator strains. Proc Natl Acad Sci. 2014;111:1897–1902. doi:10.1073/pnas.1314423111 24449905 PMC3918763

[23] van Rhijn N, Furukawa T, Zhao C, et al. Development of a marker-free mutagenesis system using CRISPR-Cas9 in the pathogenic mould *Aspergillus fumigatus*. Fungal Genet Biol. 2020;145:103479. doi:10.1016/j.fgb.2020.103479 33122116 PMC7768092

[24] Zhao C, Fraczek MG, Dineen L, et al. High-throughput gene replacement in *Aspergillus fumigatus*. Curr Protoc Microbiol. 2019;54:e88. doi:10.1002/cpmc.88 31518064 PMC9286431

[25] Li H. (2013). Aligning sequence reads, clone sequences and assembly contigs with BWA-MEM.

[26] Li H, Handsaker B, Wysoker A, et al. The sequence alignment/Map format and SAMtools. Bioinformatics. 2009;25:2078–2079. doi:10.1093/bioinformatics/btp352 19505943 PMC2723002

[27] Institute B. 2019. Picard Toolkit.

[28] McKenna A, Hanna M, Banks E, et al. The genome analysis toolkit: A MapReduce framework for analyzing next-generation DNA sequencing data. Genome Res. 2010;20:1297–1303. doi:10.1101/gr.107524.110 20644199 PMC2928508

[29] Van der Auwera GA, Carneiro MO, Hartl C, et al. From FastQ data to high confidence variant calls: the genome analysis toolkit best practices pipeline. Curr Protoc Bioinforma Ed Board Andreas Baxevanis Al. 2013;43:11.10.1-11.10.33. 10.1002/0471250953.bi1110s43 PMC4243306 25431634

[30] Smit A, Hubley R, Green P. (2015). RepeatMasker Open-4.0. 2013–2015.

[31] Cingolani P, Platts A, Wang le L, et al. A program for annotating and predicting the effects of single nucleotide polymorphisms, SnpEff: SNPs in the genome of Drosophila melanogaster strain w 1118; iso-2; iso-3. Fly (Austin). 2012;6:80–92. doi:10.4161/fly.19695 22728672 PMC3679285

[32] Tisi R, Vertemara J, Zampella G, et al. Functional and structural insights into the MRX/MRN complex, a key player in recognition and repair of DNA double-strand breaks. Comput Struct Biotechnol J. 2020;18:1137–1152. doi:10.1016/j.csbj.2020.05.013 32489527 PMC7260605

[33] Álvarez-Escribano I, Sasse C, Bok JW, et al. Genome sequencing of evolved aspergilli populations reveals robust genomes, transversions in *A. flavus*, and sexual aberrancy in non-homologous end-joining mutants. BMC Biol. 2019;17:1–17. doi:10.1186/s12915-019-0702-0 31711484 PMC6844060

[34] Dos Reis TF, Silva LP, de Castro PA, et al. The influence of genetic stability on *Aspergillus fumigatus* virulence and azole resistance. G3 (Bethesda). 2018;8:265–278. doi:10.1534/g3.117.300265 29150592 PMC5765354

[35] Zha S, Boboila C, Alt FW. Mre11: roles in DNA repair beyond homologous recombination. Nat Struct Mol Biol. 2009;16:798–800. doi:10.1038/nsmb0809-798 19654615

[36] LamVmens K, Bemeleit DJ, Möckel C, et al. The Mre11:Rad50 structure shows an ATP-dependent molecular clamp in DNA double-strand break repair. Cell. 2011;145:54–66. doi:10.1016/j.cell.2011.02.038 21458667 PMC3071652

[37] Auxier B, Debets AJM, Stanford FA, et al. The human fungal pathogen *Aspergillus fumigatus* can produce the highest known number of meiotic crossovers. PLoS Biol. 2023;21:e3002278. doi:10.1371/journal.pbio.3002278 37708139 PMC10501685

