# Supplementary material for: Accelerated mutator phenotype in a clinical Aspergillus fumigatus isolate contributes to adaptive evolution
Source: Emerg Microbes Infect. 2026 Mar 16;15(1):2627078. doi: 10.1080/22221751.2026.2627078 (PMC12997362; doi:10.1080/22221751.2026.2627078)

Supplemental tables and figures

Supplemental table 1. Mutation rate calculation and data on cell lengths

Isolate	1	2	3	4	5	6	7	8	9	10	Total distance (mm)	# estimated number of cells produced (cell equivalents)	AVG	AVG SNPs	Mutation rate
130-14: 1	18	31	17	37	36	30	18	29	47	22	285	6555			
130-14: 2	22	38	28	45	44	32	33	33	49	21	345	7935	7115	12	5.73^{-11}
130-14: 3	17	29	28	30	44	25	30	32	43	20	298	6854			
147-03: 1	60	23	37	43	27	44	40	41	47	21	383	8809			
147-03: 2	60	24	40	41	20	45	38	22	35	32	357	8211	8725	0.33	1.29^{-12}
147-03: 3	60	30	49	35	21	41	39	45	43	35	398	9154			
155-40: 1	60	60	15	53	48	25	49	50	50	50	460	10580	10258	0	-
155-40: 2	60	60	52	48	46	19	50	34	49	48	466	10718			

155-40: 3	60	60	12	52	19	30	51	45	44	39	412	9476

Measurements of growth per week in mm

Supplemental table 2. gRNA and primers to generate F332L transformants.

**Name**	**Sequence (5'-3')**	**Application**
mre11_crRNA_F332L_up	ACTTAAGCTCTTGCTGCTCTT	Forward guide RNA for generation of F332L
mre11_crRNA_F332L_down	ACATGGCGTTCAGGCAATTGC	Downstream guide RNA for generation of F333L
mre11_F	CACACACTTAAGCTCTTGCTGCTCTTTCGCACTTTAAGAAGTCCA	Forward primer to generate the front half of repair template
mre11_R	ATTGCCTGAACGCCATGTAGATGCAATACAGCTGATAAGACGCTC	Reverse primer to generate the front half of repair template
Hgy_F	ACATGGCGTTCAGGCAATACGGCGTAACCAAAAGTCACACAACACA	Forward primer to generate the second half of repair template containing HgyR

Hgy_R	TTCCTTAAACCGTAAGACCTGCATCTTGACGACCGTTGATCTGCTT	Reverse primer to generate the second half of repair template containing HgyR
2Temp_F	AGCAGTTGTCCTCCGTAGTTCACACACTTAAGCTCTTGCT	Forward primer to generate the whole length of repair template
2Temp_R	GTTGTGAAGAATGGCTGAAATTCCTTAAACCGTAAGACCT	Reverse primer to generate the whole length of repair template
PCR_Front_F1	TGGCTGTGTTTGTTGTCTCC	PCR for examination of recombination
PCR_Front_R1	CTTGTCACCCAAGCAGTTCA	PCR for examination of recombination
PCR_Mid_F2	CTGGTCGGGAGTTCAAATGT	PCR for examination of recombination
PCR_Mid_R2	AGCTGCTCCACCTTGACTGT	PCR for examination of recombination
PCR_Back_F	GCTTTCGAGCCTATGCAGAC	PCR for examination of recombination
PCR_Back_R	ATCTACGGGCTGTGCATTTC	PCR for examination of recombination

Supplemental table 3. Each parental isolate is marked in grey

Isolate	Timepoint	STRaF3A	STRaF3B	STRaF3C	STRaF4A	STRaF4B	STRaF4C
V130-14	T=0	22	6	14	6	10	5
V130-14-R1	T=10	23	6	15	5	9	5
V130-14-R2	T=10	23	6	15	6	9	5
V130-14-R3	T=10	23	6	14	6	9	5
V147-03	T=0	23	6	14	6	5	5
V147-03-R1	T=10	25	7	15	5	3	4
V147-03-R2	T=10	26	8	15	5	4	5
V147-03-R3	T=10	23	6	14	5	4	4
V155-40	T=0	32	6	8	5	7	18
V155-40-R1	T=10	31	5	6	5	6	17

V155-40-R2	T=10	31	5	6	4	6	15
V155-40-R3	T=10	31	5	6	4	6	16

Supplemental Figure 1

Typical growth of hyphal cells, observed and measured after Blancophor staining under fluorescence microscopy, 200x magnification. A total of 134 cells were measured and the average cell length was calculated to be 44 µm.

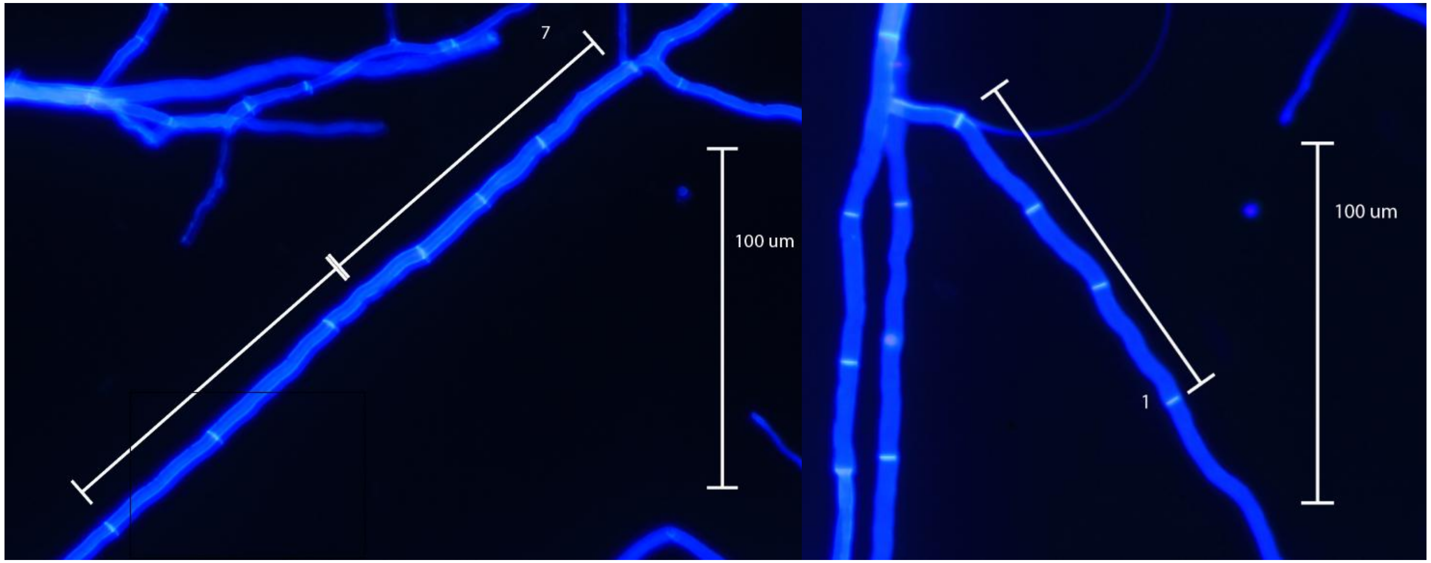

Supplemental Figure 2: Comparison of growth rates of recombinant and mutator strain

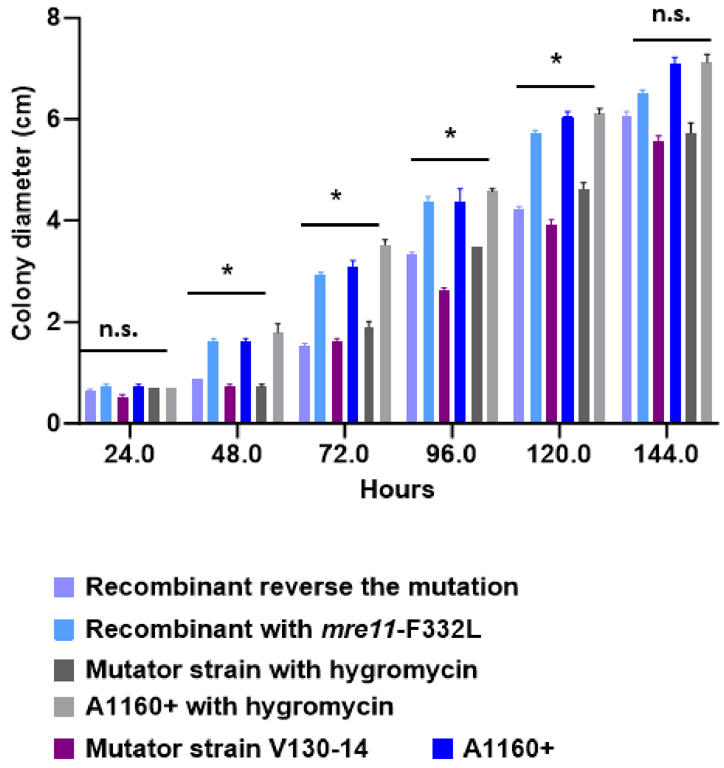

Supplement: Supplementary materials 12_12_2025.pdf [file TEMI_A_2627078_SM5892.pdf]
